# Mining the Micro-Trajectory of Two-Wheeled Non-Motorized Vehicles Based on the Improved YOLOx

**DOI:** 10.3390/s24030759

**Published:** 2024-01-24

**Authors:** Dan Zhou, Zhenzhong Zhao, Ruixin Yang, Shiqian Huang, Zhilong Wu

**Affiliations:** 1 School of Architecture and Transportation Engineering, Guilin University of Electronic Technology, Guilin 541004, China; zhenzhongz@mails.guet.edu.cn (Z.Z.); ruixin_yang@mails.guet.edu.cn (R.Y.); huangshiqian@mails.guet.edu.cn (S.H.); 791105165@mails.guet.edu.cn (Z.W.); 2 Guangxi Key Laboratory of ITS, Guilin University of Electronic Technology, Guilin 541004, China; 3 Department of Science and Technology of Guangxi Zhuang Autonomous Region, Guilin 541004, China

**Keywords:** intelligent transportation system, vehicle detection, object tracking, trajectory extraction, data mining

## Abstract

Two-wheeled non-motorized vehicles (TNVs) have become the primary mode of transportation for short-distance travel among residents in many underdeveloped cities in China due to their convenience and low cost. However, this trend also brings corresponding risks of traffic accidents. Therefore, it is necessary to analyze the driving behavior characteristics of TNVs through their trajectory data in order to provide guidance for traffic safety. Nevertheless, the compact size, agile steering, and high maneuverability of these TNVs pose substantial challenges in acquiring high-precision trajectories. These characteristics complicate the tracking and analysis processes essential for understanding their movement patterns. To tackle this challenge, we propose an enhanced You Only Look Once Version X (YOLOx) model, which incorporates a median pooling-Convolutional Block Attention Mechanism (M-CBAM). This model is specifically designed for the detection of TNVs, and aims to improve accuracy and efficiency in trajectory tracking. Furthermore, based on this enhanced YOLOx model, we have developed a micro-trajectory data mining framework specifically for TNVs. Initially, the paper establishes an aerial dataset dedicated to the detection of TNVs, which then serves as a foundational resource for training the detection model. Subsequently, an augmentation of the Convolutional Block Attention Mechanism (CBAM) is introduced, integrating median pooling to amplify the model’s feature extraction capabilities. Subsequently, additional detection heads are integrated into the YOLOx model to elevate the detection rate of small-scale targets, particularly focusing on TNVs. Concurrently, the Deep Sort algorithm is utilized for the precise tracking of vehicle targets. The process culminates with the reconstruction of trajectories, which is achieved through a combination of video stabilization, coordinate mapping, and filtering denoising techniques. The experimental results derived from our self-constructed dataset reveal that the enhanced YOLOx model demonstrates superior detection performance in comparison to other analogous methods. The comprehensive framework accomplishes an average trajectory recall rate of 85% across three test videos. This significant achievement provides a reliable method for data acquisition, which is essential for investigating the micro-level operational mechanisms of TNVs. The results of this study can further contribute to the understanding and improvement of traffic safety on mixed-use roads.

## 1. Introduction

As China’s urbanization accelerates, it is accompanied by the escalating challenges of traffic congestion and safety concerns. Within this context, two-wheeled non-motorized vehicles (TNVs) have risen to prominence as a primary transportation mode for urban dwellers. Their appeal largely stems from the low travel costs and flexibility they offer, especially on the flat roads of the southern cities of China. According to publicly available statistics, by the end of 2021 the total number of electric bicycles in China had surpassed 340 million [1]. This statistic is expected to increase because of their low cost and ease of use. However, this could also worsen traffic safety risks on mixed-use roads. Incidents such as running red lights, speeding, and hit-and-run occurrences have become increasingly frequent [2,3,4]. Data from the National Bureau of Statistics indicates that in 2021 there were a total of 35,141 traffic accidents involving TNVs nationwide [5]. This statistic reflects a staggering 128% increase in the accident occurrence rate compared to the year 2015. Therefore, it becomes essential to examine the behavioral characteristics of TNVs in order to understand their operational mechanisms and inform decision-making processes in traffic safety, management, and control. Nonetheless, achieving a comprehensive understanding of TNV behavior and associated accident risks remains challenging, largely due to limitations in data collection.

Driving trajectories, which encompass both temporal and spatial information, are crucial for studying the behavioral characteristics of vehicles [6]. The effective utilization of spatio-temporal data to extract the potential operating characteristics of targets can provide robust data support for the prevention and management of traffic safety issues. For instance, within an intelligent networked environment, self-driving cars leverage spatio-temporal data to perceive the actions of nearby vehicles and adapt their driving strategies accordingly. This approach significantly contributes to the reduction in traffic congestion [7]. Furthermore, forecasting the movement of people using spatio-temporal data can assist in proactively managing traffic in critical areas [8]. In a similar vein, if high-quality trajectory data for TNVs can be effectively obtained, it can provide invaluable support for comprehensively understanding the microscopic movement patterns of TNVs. This knowledge could then be instrumental in guiding road traffic safety, particularly in areas without mixed traffic.

One of the most prevalent trajectory datasets is the Next Generation Simulation (NGSIM) dataset, which was introduced by the United States Federal Highway Administration [9]. NGSIM is compiled through the processing of traffic videos captured using cameras mounted on roadside buildings, encompassing sections of both highways and urban streets. It includes continuous trajectories of motor vehicles, along with details like vehicle speeds, accelerations, coordinates, and types. However, the NGSIM dataset lacks data pertaining to TNVs. Furthermore, it is constrained by the positioning of fixed cameras, which results in coverage of only fixed road segments, limited overall coverage, and a singular vehicle type focus.

In recent years, the swift advancement of unmanned aerial vehicle (UAV) technology has offered a novel method for trajectory data acquisition. Its benefits include lower costs and expansive aerial perspectives, yielding trajectory data characterized by enhanced continuity and precision. UAVs have, thus, emerged as the mainstream medium for trajectory data collection. Nonetheless, when compared to motor vehicles like cars and buses, TNVs often present challenges due to their smaller size, higher density, and less distinctive features in aerial videos, complicating their accurate detection and rapid identification. Additionally, the process of aerial photography introduces complexities due to the simultaneous motion of both the target and the UAV, making the positioning task more challenging. This paper introduces a methodological framework designed for mining micro-trajectories of TNVs in urban road environments. The key contributions of this work are outlined as follows:

1. We propose a novel method for mining TNVs trajectory data, utilizing the You Only Look Once Version X (YOLOx) [10] target detection model coupled with the Deep Sort multi-target tracking algorithm. This approach facilitates frame-by-frame vehicle location tracking. The selection of YOLOx is a comprehensive outcome, balancing between model detection performance and model size.

2. We propose the integration of a Convolutional Block Attention Mechanism (CBAM) based on median pooling into the YOLOX framework, specifically designed to detect TNVs with small-sized targets. This enhancement significantly improves the model’s detection performance for small target vehicles.

3. We employ a range of techniques, including video stabilization, coordinate mapping, and filter denoising, to accurately reconstruct the vehicle’s motion trajectory.

The remainder of this paper is organized as follows: Section 2 provides a review of research on mining vehicle trajectories and analyzes efforts to enhance detection performance for small target objects. Section 3 describes the specific methodology and experimental program and Section 4 presents the experiment results and analyzes them objectively.

## 2. Related Works

The evolution of urban transportation in China began relatively late, leading to a conspicuous discrepancy and imbalance between the advancement of transportation management and the pace of rapid urbanization. Notably, TNVs present significant challenges to urban traffic management, primarily due to their inherent mobility and flexibility. Exploring the microscopic behavioral characteristics of TNVs and conducting in-depth analysis of their microscopic traffic mechanisms have emerged as pressing issues for many scholars and city managers. However, due to constraints in data acquisition, existing research has not been able to fully capture the microscopic driving behavior characteristics of non-motor vehicles. Certain studies have utilized GPS data from shared bicycles and delivery workers [11,12,13], but this data tends to be of coarse granularity and is limited by the issue of a single type of sample. Utilizing computer vision technology to extract vehicle trajectories from aerial videos represents a highly efficient method. However, the TNVs examined in this study are characterized by their small size and high density in high-altitude videos, which presents significant challenges for feature extraction. Numerous studies have focused on video-based trajectory extraction of medium and large vehicles, such as cars, but have neglected small targets like TNVs. This section will review research related to video trajectory extraction and efforts to enhance recognition performance for small targets. The related work and methodologies also provide a substantial reference foundation for this article.

### 2.1. Progress of Trajectory Data of Drone Aerial Photography 

Compared to the traditional methods of manual statistics and camera-based data capture for acquiring micro-traffic flow data, the emerging approach that combines UAV aerial photography with computer vision has garnered widespread attention from researchers due to its high efficiency and low error rate. Liu et al. [14] utilized UAV to collect traffic videos in urban highway exit areas and proposed a composite framework to extract vehicle trajectories. This framework integrates the YOLOv4 vehicle detection algorithm, SORT vehicle tracking algorithm, and the KD-tree trajectory data reconstruction algorithm. Experiments demonstrate that this framework can effectively capture most car trajectories. However, the SORT algorithm struggles with effectively handling obstructions in the background. Lin et al. [15] proposed a method for acquiring traffic data, such as vehicle flow and speed at intersections, using aerial videos captured using unmanned aerial vehicles. This method employs background subtraction and image morphological operations to detect vehicles in the video and utilizes the KCF algorithm for tracking vehicle targets to extract their trajectories. However, this method lacks the ability to distinguish between different types of vehicles, resulting in all moving objects being recorded indiscriminately. To identify the formation and triggering factors of congestion bottlenecks in fast road weaving areas in real-time, Li et al. [16] constructed vehicle trajectory data based on aerial videos captured using UAVs. They proposed a congestion identification method for weaving areas, incorporating an analysis of unstable traffic flow. However, the effectiveness of this method has not been confirmed in mixed traffic sections involving both motorized and non-motorized vehicles. Wang et al. [17] utilized target detection algorithms and Kalman filtering to capture and analyze traffic flow from unmanned aerial vehicle videos. However, Kalman filtering is notably sensitive to initial values, which may significantly impact the results. Li et al. [18] developed an adaptive framework for estimating the ground speed of multiple vehicles in UAV aerial videos. They proposed a homography-based motion compensation method to determine the actual motion trajectories of vehicles in the current video frame. This method necessitates the real dimensions of the cars as prior information for guidance.

Relevant literature indicates that using UAVs to acquire traffic flow data is both feasible and reliable. The implementation primarily involves two steps. First, based on the principles of computer vision, feature extraction is conducted in aerial videos to detect and recognize vehicles. Subsequently, target tracking of the detected vehicles is achieved through relevant filtering algorithms and deep learning algorithms. However, the aforementioned methods have not been validated in urban road sections with mixed motorized and non-motorized traffic. Moreover, the focus of trajectory extraction has been limited to cars, without consideration for other types of vehicles, such as TNVs.

### 2.2. Small Target Detection

The TNVs analyzed in this paper are characterized by their small size, dense grouping, and blurred features in aerial videos, which pose significant challenges for their detection and recognition. Efficiently detecting small targets against a large background has emerged as a current research hotspot. Presently, research on small target detection primarily revolves around the utilization of feature pyramid networks, attention mechanisms, and their collaborative integration. Some researchers have enhanced feature extraction for small targets by improving residual structures and adding multi-branch cross-scale modules in feature pyramid networks [18,19,20]. However, this also implies increased model complexity, leading to excessive consumption of computational resources. In the realm of attention mechanisms, Cao et al. [21] introduced a multi-dimensional attention gate network by integrating spatial, channel, and multi-dimensional feature map inputs. This network captures the global distribution of semantic information across spatial and channel dimensions, facilitating enhanced feature response. Wang et al. [22] developed a bidirectional attention network by merging multi-channel attention modules and multi-attention fusion modules. This network provides an abundance of information for the feature fusion of small targets. However, overly complex attention mechanisms may lead to overemphasis on certain features while neglecting other crucial ones, potentially resulting in decreased performance. Many studies have integrated both approaches to augment the feature extraction capabilities for small objects. For instance, in studies [23,24,25], researchers have enhanced the network’s perception of fine details on small objects by combining various attention mechanisms within the feature pyramid network, yielding promising results. However, this does not imply that a more complex model is necessarily better. On the contrary, more intricate models require substantial prior knowledge to fine-tune their hyperparameters, and they also tend to reduce the detection speed of the model. Additionally, some researchers have approached small target detection from alternative perspectives. For instance, Bosquet et al. [26] introduced a data augmentation method that combines generative adversarial networks (GANs) to achieve high-quality data synthesis. However, it is important to note that excessive data augmentation can introduce more noise. This makes it more susceptible to overfitting. Kim et al. [27] introduced a novel loss function based on the Wasserstein distance to specifically address the detection of small target objects. This approach effectively overcomes issues related to sensitivity of intersection and union in narrow scenarios. Although the method is effective for detecting small targets, it may be necessary to use a larger sample size for more accurate learning and evaluation. Shan et al. [28] designed a bidirectional feedback framework (GKB) that optimizes the interplay between Gaussian mixture models and kernelized correlation filters to enhance vehicle detection. The method also places a greater computational burden on the model.

In summary, existing trajectory extraction methods are primarily tailored for medium to large targets such as cars, with limited research focused on the trajectory mining of TNVs. Additionally, in the realm of small target detection, there is a lack of an effective balance between detection performance and the consumption of computational resources.

## 3. Methods

**Figure 1:**
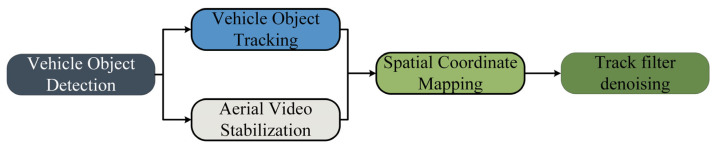
Overview of the framework structure.

The primary objective of this paper is to construct a methodological framework for extracting the trajectories of TNVs at urban road intersections and mixed traffic flow bottleneck segments. Our study focuses on non-motorized vehicles, which are often overlooked in traditional trajectory extraction studies. This class of vehicles presents a challenge due to their small size, and our research aims to address this lack in the field of mining TNV trajectories. The data obtained can be utilized to study the microscopic driving behavior of non-motorized vehicles, addressing the current challenge of acquiring fine-grained trajectory data for these vehicles. The framework consists of five modules: vehicle object detection, vehicle object tracking, aerial video stabilization, spatial coordinate mapping, and trajectory filtering denoising. The overall structure of the framework is shown in Figure 1. In the video stabilization phase, the optical flow method is employed to perform feature-based stabilization of UAV aerial videos, resulting in a stable video for subsequent detection. In the vehicle target detection module, an optimized and improved single-stage object detection algorithm YOLOx is used as the vehicle target detector to obtain the vehicle bounding boxes. Subsequently, the Deep Sort multi-object tracking algorithm is employed to track the successfully detected vehicles. Vehicle target detection and target tracking work simultaneously to form the initial vehicle trajectories. After obtaining the coordinate location information of vehicles, pixel coordinates are transformed into real-world physical coordinates, and vehicle coordinates are correlated to obtain a continuous trajectory. Finally, Kalman filtering is applied to denoise the trajectory, eliminating errors and improving trajectory quality.

### 3.1. Improved YOLOx Model

**Figure 2:**
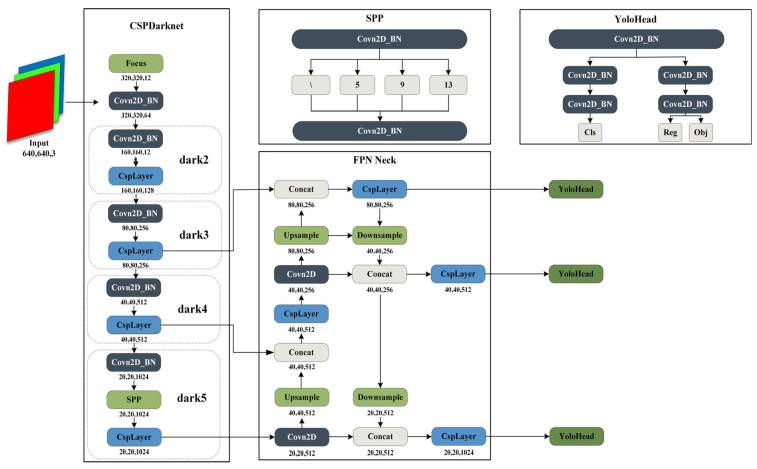
YOLOx is composed of four parts: input, backbone, neck, and head.

YOLOx [10], initially introduced by Megvii Technology in 2021, falls within the YOLO series of single-stage detection algorithms. In contrast to multi-object detection algorithms such as YOLOv3 [29] and YOLOv5 [30], YOLOx distinguishes itself by integrating several noteworthy techniques, including SimOTA (dynamic sample matching), decoupled detection heads, and the incorporation of the Focusstructure. The network architecture is illustrated in Figure 2.

#### 3.1.1. Convolutional Block Attention Module Based on Median Pooling 

**Figure 3:**
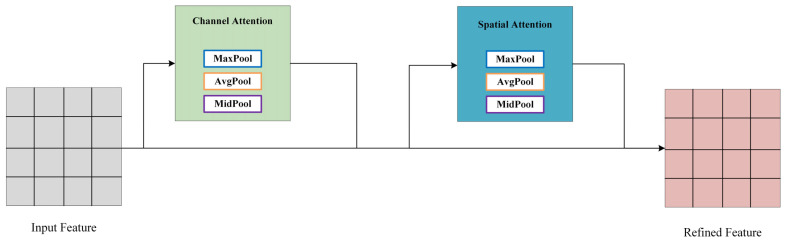
M-CBAM generates features through the combined action of a channel attention mechanism and a spatial attention mechanism.

Convolutional Block Attention Module (CBAM) is a lightweight attention module designed for feedforward convolutional neural networks [31]. This section introduces an enhancement to CBAM, presenting a median pooling-based CBAM (M-CBAM), as shown in the structure diagram in Figure 3.

The M-CBAM introduced in this paper comprises a channel attention module and a spatial attention module. The output of the convolutional layer initially passes through the channel attention module to obtain weighted results and subsequently enters the spatial attention module, ultimately resulting in the overall weighted output.

**Figure 4:**
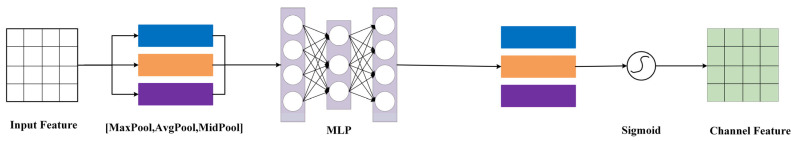
Median pooling layer embedded in the channel attention module.

The channel attention module processes the input feature map by applying global max pooling, global average pooling, and global median pooling based on width and height. The resulting features are then fed into a fully connected neural network to obtain output features, which are subsequently activated using the sigmoid function. The channel attention features are illustrated in Figure 4 and expressed using the following equation:

(1) $$\begin{array}{ll} M_{c}(F)= & \sigma (MLP(AvgPool(F))\\ & +MLP(MaxPool(F)\\ & +MLP(MidPool(F)) \end{array}$$

where $M_{c}$ represents the channel attention map; F represents the intermediate feature map; σ represents the sigmoid function; MLP represents the multilayer perceptron; AvgPool denotes average pooling; MaxPool denotes maximum pooling; and MidPool denotes median pooling.

**Figure 5:**
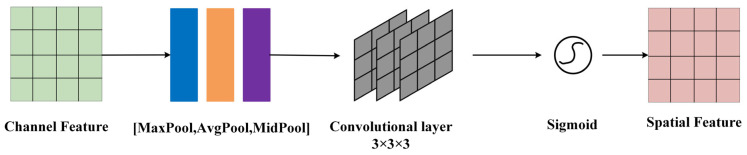
Median pooling implanted in the spatial attention module and replacing the convolutional layer.

The input of the spatial attention module consists of channel attention features and image input features. First, the input features undergo channel-based max pooling, average pooling, and median pooling. The pooled features are stacked and then subjected to dimension reduction through three layers of convolutional operations. The resulting feature is activated through the sigmoid function to obtain the final spatial attention feature as shown in Figure 5, represented as follows:

(2) $$\begin{array}{ll} M_{s}(F)= & \sigma (f^{3\times 3}(f^{3\times 3}(f^{3\times 3}(\\ & [AvgPool(F);\\ & MaxPool(F);\\ & MidPool(F)])))) \end{array}$$

where $M_{s}$ represents the channel attention map; F represents the intermediate feature map; σ represents the sigmoid function; MLP represents the multilayer perceptron; AvgPool denotes average pooling; MaxPool denotes maximum pooling; and MidPool denotes median pooling.

The proposed median pooling in this article possesses the following characteristics: compared to max pooling and min pooling, median pooling exhibits stronger noise resistance, enabling better exploration of target and background features in image information; simultaneously, median pooling can fully utilize spatial and temporal neighboring information in images, especially for closely located small target objects, showing excellent resolution and recognition effects.

The M-CBAM proposed in this paper, based on the original model, incorporates a median pooling layer into the channel attention module and spatial attention module. This addition effectively enhances the network’s capability to learn target edges and texture structure characteristics, while also improving noise resistance. Furthermore, in the channel attention module, the original single 7 × 7 convolution is replaced with three layers of 3 × 3 convolutions, reducing model parameters and saving computational costs.

#### 3.1.2. The Fourth Small Object Detection Head

The design of decoupled detection heads improves the performance of YOLOx in terms of bounding box regression and target classification, but it does not directly benefit the localization of small objects. Additionally, YOLOx may suffer from feature loss of small target objects when used in conjunction with downsampling layers for feature extraction. Consequently, to improve the model’s detection performance for small objects, an additional small target detection head connected to the backbone network has been incorporated into the network architecture. This additional detection head predicts on larger feature maps and retains more small target objects, partially compensating for the feature loss that occurred during the small target feature extraction in the original network. However, this does not imply that more detection heads are better, as additional detection heads increase the model’s computational burden. In this paper, the addition of only one extra detection head is a balanced consideration of model performance and computational cost.

#### 3.1.3. Loss Function Optimization

The original YOLOx uses IoU [32] loss to measure the overlap between the ground truth box and the predicted box, which is the intersection over union (IoU). However, when the predicted box and the ground truth box do not intersect, the IOU is 0, which cannot reflect the distance between the two anchor boxes. In this case, the loss function becomes non-differentiable, leading to gradient vanishing issues, preventing the neural network from further learning. The formula for calculating IOU is as follows:

(3) $$IoU=\frac{\left|A\cap B\right|}{\left|A\cup B\right|}$$

where A represents the predicted box and B represents the ground truth box.

Based on the issues with the IoU loss mentioned above, this paper introduces the CIoU [33] loss as an improvement over the IoU loss in the model. It incorporates penalties for both the distance between the predicted box and the ground truth box centers and aspect ratio in addition to considering the overlap area between the prediction and the actual bounding boxes. The formula for calculating CIoU loss is as follows:

(4) $$CIoU=1-IoU+\frac{\rho (b,b^{gt})}{c^{2}}+\alpha v$$

(5) $$\alpha =\frac{v}{(1-IoU)+v}$$

(6) $$v=\frac{4}{\pi^{2}}{\left(\arctan\frac{w^{gr}}{h^{gt}}-\arctan\frac{w}{h}\right)}^{2}$$

(7) $$CIoU_{loss}=1-CIoU$$

Within the formula, $\rho (b,b^{gt})$ signifies the distance between the centroids of the predicted box and the ground truth box, c corresponds to the length of the diagonal of the minimum enclosing rectangle, α serves as a weighting parameter, and v is employed to quantify the consistency of the aspect ratio between the ground truth box and the predicted box.

### 3.2. Vehicle Motion Trajectory Extraction

#### 3.2.1. Vehicle Tracking Based on the Deep Sort Algorithm

To acquire trajectory information for moving vehicles, dynamic tracking and positioning of the vehicles are required. Considering the issue of occlusion caused by factors such as roadside greenery and traffic signals in urban road traffic, this paper utilizes the Deep Sort multi-object tracking algorithm to achieve the tracking and positioning of moving vehicles [34]. Deep Sort is an enhancement of the SORT object tracking algorithm, which incorporates additional appearance feature information of moving targets [35], which is characterized using the cosine distance with the following equation:

(8) $$d(i,j)=\min\{1-r_{j}^{T}r_{k}^{i}|r_{k}^{i}\in R^{i}\}$$

where d(i,j) denotes the minimal cosine distance between the appearance features, $r_{j}^{T}r_{k}^{i}$ denotes the cosine similarity of the appearance features between the j-th detection image and the i-th tracking image, and $R^{i}$ denotes the library of appearance features. The smaller d(i,j) represents the higher similarity of the appearance features between the tracking frame and the detection frame.

**Figure 6:**
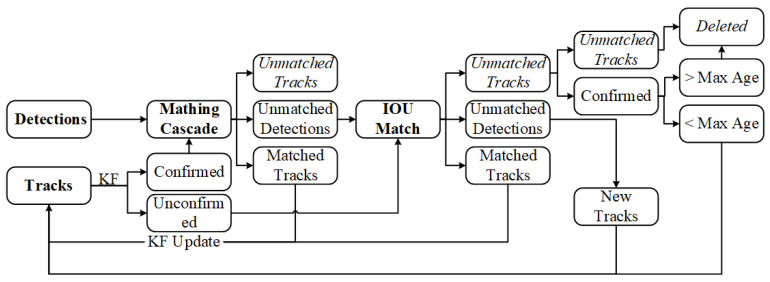
Flowchart of Deep Sort algorithm.

The tracking process of Deep Sort for vehicles is outlined in Figure 6 and consists of the following steps:

- **Object Detection:** Based on the detection results of YOLOx in the first frame of the video, corresponding initial tracks are created, and the Kalman filter is used to predict the potential locations of vehicle targets in the second frame of the image.
- **Cascade Matching:** In the second frame, predicted trajectories are divided into two states: confirmed state (CS) and unconfirmed state (US). CS is considered as a vehicle target, while the US is uncertain whether it is a vehicle target. CS is associated with the detection results in the second frame, taking into account the motion state and appearance of CS and whether they match the detection box. The matching method uses cascade matching, resulting in three preliminary outcomes for the second frame: matched tracks, unmatched tracks, and unmatched detections. Among these, the matched tracks are directly updated with Kalman filtering into the vehicle target trajectories, while unmatched track boxes and unmatched detection boxes represent matching failures and await further processing.
- **IoU Matching:** The unmatched track boxes and unmatched detection boxes mentioned above are further associated using IoU matching, where a higher IoU value indicates greater proximity and a higher likelihood of belonging to the same vehicle target. By setting an IoU threshold, three outcomes are filtered: matched tracks, unmatched track boxes, and unmatched detection boxes. Among these, the matched tracks are updated into vehicle target trajectories through Kalman filtering. The further filtered unmatched track boxes and unmatched detection boxes await subsequent processing.
- **Processing of Unmatched Detection Boxes:** For the remaining unmatched detection boxes, new trajectories must be reconstructed. Simultaneously, these newly established trajectories are confirmed. If they are determined to be vehicle targets and not other objects, they are likewise updated into the existing vehicle target trajectories.
- **Processing of Unmatched Track Boxes:** Due to missed detections in the detection stage, situations with unmatched track boxes can occur. It is necessary to confirm the unmatched track boxes. If they do not represent vehicle targets, they are deleted. If they are identified as vehicle targets, they are initially retained. Within 30 frames, if they successfully match with detection boxes in subsequent frames, they are updated into the vehicle target trajectories. If no successful match occurs, they are deleted. This process is repeated to achieve multi-object tracking in the video stream.

#### 3.2.2. Aerial Video Stabilization

During high-altitude video capture using UAV, the action of propellers and external wind forces can disrupt the stability of the aircraft, leading to video jitter. Such jitter can lead to drifting in the relative positions of vehicles, hindering vehicle tracking and the subsequent extraction of trajectories. This section aims to stabilize drone aerial videos through a method based on feature points. The specific procedures include:

(1) Input the video for detection and identify the image’s feature points.

(2) Utilize optical flow to track the image’s feature points and construct a motion affine transformation matrix based on the alterations in feature points between the previous and current frames.

(3) Calculate motion trajectories using the motion affine transformation matrix and apply motion trajectory smoothing.

(4) Generate a smoothed affine transformation matrix based on the smoothed motion trajectories.

(5) Ultimately, the stabilized video image is produced through the smoothed affine transformation matrix.

#### 3.2.3. Spatial Coordinate Mapping

Initially, the camera coordinate system (X,O,Y,Z) and image coordinate system (x,o,y) are established. Through the pinhole imaging principle, the camera coordinate system can be projected onto the image coordinate system.

Subsequently, the pixel coordinate system (u,o,v) is established, and the following relationship exists between the image coordinate system (x,o,y) and the pixel coordinate system (u,o,v):

(9) $$\left\{\begin{array}{l} u=f_{x}x+c_{x}\\ v=f_{y}y+c_{y} \end{array}\right.$$

where $f_{x}$ represents the scaling factor for pixel coordinates along the u-axis, $f_{y}$ represents the scaling factor for pixel coordinates along the v-axis, and $\left(c_{x},c_{y}\right)$ denotes the pixel coordinates of the image plane center in the pixel coordinate system. The equation can be rewritten in matrix multiplication form as follows:

(10) $$\left[\begin{array}{c} u\\ v\\ 1 \end{array}\right]=K\left[\begin{array}{c} x\\ y\\ 1 \end{array}\right]$$

where K represents the camera’s intrinsic matrix, which can be obtained by referring to the drone’s specification parameters.

### 3.3. Experimental Arrangements

#### 3.3.1. Data Source 

**Figure 7:**
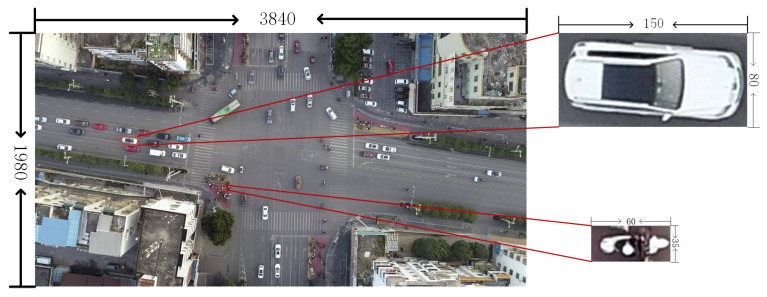
The size of the two-wheeled non-motorized vehicle target in the dataset is 60 × 35 pixels, which is much smaller than the automobile target.

During the research expedition in cities Guilin and Nanning, China, the team identified road segments and intersections with expansive visibility and limited roadside obstructions for capturing aerial videos. The UAV used was the DJI Mavic 3, with a video frame rate of 60 fps and dimensions of 3840 × 1980 pixels, recording at altitudes ranging from 70 m to 80 m. Frame extraction is performed on the recorded video to obtain images for network training. Image annotation for object recognition is a prerequisite for model training. Image annotation for object recognition serves as a fundamental prerequisite for model training. In this experiment, a total of 515 images were annotated. Based on real road conditions, this study categorizes traffic targets into five types: non-motorized vehicles, cars, buses, trucks, and pedestrians. An example of the dataset is shown in Figure 7.

#### 3.3.2. Experimental Configuration and Evaluation Metrics

The experiments in this study were carried out on a Windows system using the Python programming language. The experimental configuration parameters are detailed in Table 1.

To validate the performance of the model in this paper, the evaluation metrics employed include recall, precision, average precision (AP), F1 score, and model parameters [36].

(11) $$Recall=\mathrm{TP}/\left(TP+FN\right)$$

(12) $$Precision=TP/\left(TP+FP\right)$$

Among these, TP represents true positive cases, indicating the number of instances where the model predicts a positive sample and it is indeed a positive sample; FP denotes false positive cases, signifying the number of instances where the model predicts a positive sample but it is actually a negative sample; and FN stands for false negative cases, denoting the number of instances where the model predicts a negative sample but it is actually a positive sample.

Precision and recall are conflicting metrics; generally, when classification confidence is high, precision tends to be high, whereas when classification confidence is low, recall tends to be high. Therefore, to provide a comprehensive evaluation of these two metrics, the F1 score is also employed to assess model performance.

(13) $$F1=2\times \frac{Precision\times Recall}{Precision+Recall}$$

AP is a metric used to measure the overall correctness of a specific category. It is calculated as the area under the precision–recall curve and represents the average precision of the model across the entire range of recall levels. A higher AP value indicates superior model performance.

(14) $$AP=\int_{0}^{1}P(R)dR$$

The precision–recall curves (PR curves) are constructed by calculating cumulative precision values of TP or FP and recall values.

#### 3.3.3. Experimental Design 

In this study, 12 experiments were conducted to assess the model’s performance. Table 2 outlines the experimental design, with experiment 1 serving as the control group, utilizing the original YOLOx model as the baseline. Experiments 2 to 5 aim to validate the effectiveness of the model improvement methods proposed in this paper, while experiments 6 to 12 provide comparisons with prevalent object detection algorithms currently in use.

## 4. Results

### 4.1. Object Detection Results

**Figure 8:**
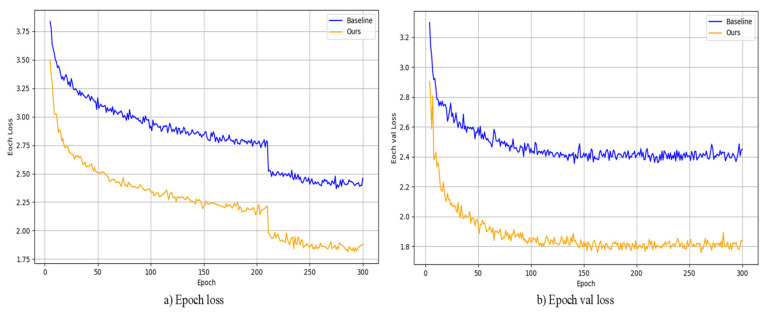
(**a**,**b**) show the loss function decreases for the baseline model and our model, respectively, and our model converges faster.

During model training, this paper employed a transfer learning strategy, using YOLOx-s as the pretrained weights and fine-tuning the model on a custom dataset. Figure 8 shows the reduction in loss during the training of the model, and the decrease in loss on the training and validation sets are shown in (a) and (b), respectively. In both sets of experiments, the loss function gradually decreases with an increase in training epochs. Compared to the baseline model, the improved model in this paper exhibits faster loss reduction and reduced oscillation in the loss curve, resulting in improved network convergence speed.

Table 3 presents the enhancement effects of the improvement strategies proposed in this paper on model performance. From experiment 2 to experiment 5, the improvement strategies introduced in this paper were gradually integrated for validation. It is noteworthy that the main focus of this study is TNVs. Additionally, due to the scarcity of buses and trucks in actual road scenarios, the model’s performance evaluation is conducted only with TNVs and passenger cars as representatives. In experiment 2, after incorporating CIoU, the model showed improvements in various evaluation metrics for both TNVs and cars. In experiment 3, the introduction of CBAM led to enhancements in AP, recall, and F1 for TNVs, but resulted in a decline for cars. In experiment 4, after incorporating the proposed M-CBAM, it compensated for the decline in car evaluation metrics observed in experiment 3 while also improving the metrics for TNVs. Experiment 5 incorporates all the improvement strategies from this paper and represents the best performing group among the five sets of experiments. Compared to the baseline model, experiment 5 achieved a 2.34% increase in recall and a 1.56% increase in precision for TNVs, as well as a 4.28% increase in recall and a 2.22% increase in precision for automobiles. The AP for TNVs reached 64.26%, showing a growth of 1.58%, while the AP for automobiles improved by 3.13%. The improvement strategies in this paper effectively enhance the model’s detection performance for small TNV targets.

In order to evaluate the improvement strategies in this paper, performance comparisons were conducted with prevalent object detection models currently in use. Table 4 displays the performance comparison results between the model introduced in this paper and the prevalent object detection models in current use. It can be seen that the baseline model before the improvement of the TNVs detection on the recall reached the best among other algorithms in the same category, in addition to its smaller model parameters and faster detection speed, which also provides guidance for us to choose YOLOx as the baseline model. Remarkably, the model in this paper achieved the best performance in both the AP and recall metrics for TNVs. In terms of model parameters, the improvement strategies in this paper have only increased by 0.83 M compared to the baseline model, which is lower than the parameters of other object detection models except YOLOv5. Detection efficiency is measured using the fps metric. The most recent YOLOv8 algorithm achieves the highest detection speed, yet its performance on TNV small targets is suboptimal. This may be attributed to YOLOv8 being pre-trained on the coco dataset, which lacks sufficient small target objects. Consequently, this affects the model’s detection rate of non-motorized vehicles. Additionally, the increased complexity of YOLOv8’s network and its frequent downsampling contribute to the loss of features of tiny targets. On the other hand, the SSD algorithm, while slightly slower in detection speed compared to YOLOv8, exhibits an exceedingly low recall on TNVs. The detection performance of the two-stage object detection algorithm Faster-RCNN is the lowest, possibly because in methods based on candidate box regression, anchor boxes correspond to the original image, and anchor boxes lose the features of small targets after multiple downsampling operations. Therefore, this leads to ineffectiveness in high-density small object detection tasks. The detection speed of our model is 28.7 FPS, which, while not the fastest, still satisfies the requirements for real-time detection. Detection speed is influenced by the complexity of the model structure. In this paper, the addition of the fourth detection head increased the size of the model and added extra parameters relative to the baseline model. This is also one of the reasons for the decrease in FPS.

**Figure 9:**
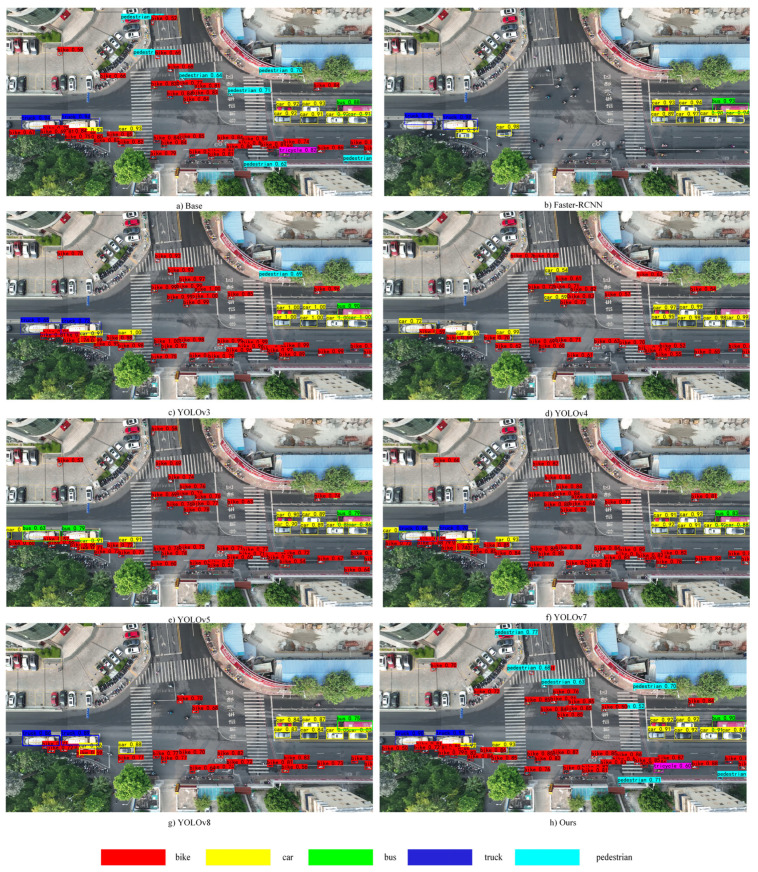
In the comparison of the detection effect with other models, our model achieves better results in terms of detection rate and correctness rate.

The detection results of different models are illustrated as shown in Figure 9. Compared to other similar object detection models, the model in this paper is capable of effectively detecting non-motorized vehicle targets, and it can even detect smaller objects such as “pedestrians”. This demonstrates the effectiveness of the improvement strategies proposed in this paper for small target objects.

### 4.2. Vehicle Motion Trajectory Extraction Results

In this section, three test videos were used to evaluate the performance of the trajectory extraction framework. In the first test video, the research team manually counted 14 (car) and 116 (bike) real trajectories; in the second video, it was 14 (car) and 102 (bike); and in the third test video, it was 25 (car) and 37 (bike). As shown in Table 5, the recall rate for car trajectories is almost the same in the three test videos, and is 92.86%, 92.86%, and 92.00%, respectively. The recall rate for bike trajectories is 81.90%, 85.29%, and 72.97%, all lower than that of cars. This is because the model performs better when detecting cars during the detection process.

In the three test videos, a total of 50 trajectories for both cars and bikes were lost or not correctly associated. Most of the lost trajectories were due to detection failures. Additionally, the research team observed that trajectory losses were lower on straight road segments like bridges compared to intersections, indicating that target density also affects trajectory construction.

**Figure 10:**
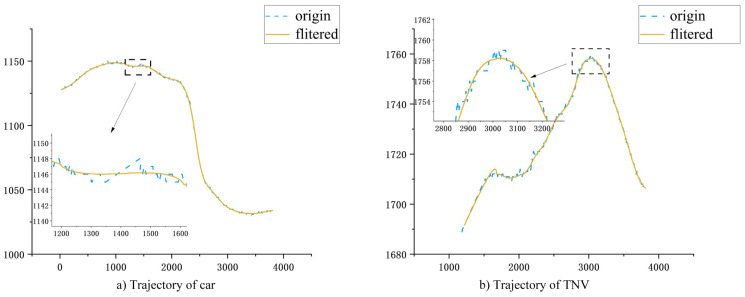
(**a**,**b**) are the results of car and TNV trajectory construction, respectively, and the trajectory extraction method in this paper can realistically restore the vehicle’s motion trajectory.

The results of denoised car and TNV trajectories are presented in Figure 10. In the coordinate plot, the blue dashed line and the orange line, respectively, represent the original trajectories and the smoothed trajectories. Figure 10 demonstrates that the smoothed trajectory data has reduced fluctuations. In fact, the trajectory construction method proposed in this paper enables frame-by-frame tracking and construction, effectively mitigating the initial trajectory data’s volatility.

## 5. Discussion

This study presents a method for extracting vehicle trajectories in dedicated non-mixed traffic lanes, thereby reducing the workload for relevant researchers. Additionally, the method can contribute to enriching trajectory data for traffic flow studies. Based on the video data from this study, more detailed research on the microscopic operational mechanisms of vehicles can be conducted, providing relevant data support for the field of traffic safety.

However, this study still has some limitations that need to be further addressed and improved by researchers, including but not limited to the following aspects:

The dataset used for model training in this study is relatively small, with an uneven sample distribution. Future research can explore the application of the current method in more diverse traffic environments, such as on different types of roads, in various cities, and even within the transportation systems of different countries. This would enable the validation of the method’s generalizability and practicality.

Considering the real-time performance and accuracy of the existing framework, future work could focus on algorithmic optimization to enhance the speed and accuracy of trajectory reconstruction. For instance, researchers could develop new algorithms to mitigate the impact of lighting variations, or employ machine learning methods to predict and correct errors in the trajectories.

Based on the extracted trajectory data, future research could delve deeper into analyzing the microscopic behavior patterns of vehicles, identifying potential risky behaviors, and providing data support for traffic safety management and urban planning.

With the rapid advancement in the field of computer vision, future research could integrate more sophisticated image processing and object recognition technologies. This includes utilizing deep learning for image segmentation and feature extraction, thereby enhancing the accuracy of small target detection.

## 6. Conclusions

This paper presents a framework for extracting vehicle trajectories based on aerial videos, particularly focusing on the trajectories of small target TNVs. The proposed framework integrates high-precision vehicle object detection, vehicle object tracking, aerial video stabilization, coordinate mapping, and trajectory filtering denoising. Testing was conducted on three different videos, and the main contributions are as follows:

(1) This paper proposes an improved YOLOx object detection method for detecting small non-motorized vehicle targets. Compared to advanced object detection algorithms, the improved YOLOx in this paper achieves the best overall detection performance for small targets such as TNVs, with an AP of 64.26% and a recall of 63.5%. Furthermore, it achieves a detection speed of 28.7 fps, meeting the requirements for real-time detection.

(2) This paper presents enhancements to the CBAM attention mechanism by incorporating median pooling layers in both the channel attention module and the spatial attention module, thereby strengthening the feature extraction capacity for small target objects. Furthermore, the original single 7 × 7 convolution layer is replaced with three layers of 3 × 3 convolution, maintaining detection performance while reducing the model parameters to 9.77 M.

(3) This paper verifies the performance when a fourth additional detection head is incorporated in the network for detecting small TNV targets. Experimental results show that after adopting the improvement strategies proposed in this paper the recall rate for TNV targets has increased by 2.43%, and the category AP has improved by 1.58%.

(4) This paper extracts vehicle trajectories based on the Deep Sort multi-object tracking algorithm. Experimental results indicate that the average recall rate of the proposed method for the trajectories of TNVs can exceed 80%.

(5) This paper has constructed a new aerial non-motorized small target dataset which contains 38,728 non-motorized small target objects, and the dataset is continuously being enriched.

The primary contribution of this study is the development of a vehicle trajectory extraction method with a specific emphasis on small non-motorized vehicle targets. This method provides an effective way to acquire data for analyzing the intricate operational mechanisms of TNVs. These vehicles pose a significant challenge due to their small size, and our research aims to bridge this gap in the domain of TNV trajectory analysis. The outcomes of this study offer substantial data support for an in-depth understanding and application of the micro-motion patterns of electric bicycles. This understanding is crucial for guiding traffic safety management in mixed traffic conditions. Additionally, the advancements achieved in detecting small targets such as TNVs in this paper can provide insights and serve as a benchmark for similar challenges in other industries or fields. 

## Figures and Tables

**Table 1:** Experimental configuration parameters.

Parameter	Configure
CPU	12th Gen Inter(R) Core(TM) i7-12700K
System	64-bit Windows System
GPU	NVIDIA GeForce RTX 3090
Programming Language	Python 3.7
Cuda Version	CUDA 11.8
Development Framework	Pytorch 1.12

**Table 2:** Experiment schedule.

No.	Model	Improvement Methods	Goals
1	YOLOx	-	Baseline
2	-	+CIoU	Our improvement
3	-	+CBAM	
4	-	+M-CBAM	
5	-	+Fourth head	
6	SSD [37]	-	Compare with others
7	Faster-RCNN [38]	-	
8	YOLOv3	-	
9	YOLOv4 [39]	-	
10	YOLOv5	-	
11	YOLOv7 [40]	-	
12	YOLOv8 [41]	-	

**Table 3:** Assessment results of improvement strategies’ effectiveness.

No.	Strategy	AP (%)		Recall (%)		F1 Score		Precision (%)	
		Bike	Car	Bike	Car	Bike	Car	Bike	Car
1	-	62.68	90.34	61.16	90.69	0.74	0.92	94.29	92.43
2	+CIoU	62.98 (↑0.30)	90.68 (↑0.34)	62.49 (↑1.33)	93.47 (↑2.78)	0.75	0.93	94.74 (↑0.65)	93.32 (↑0.89)
3	+CBAM	63.13 (↑0.05)	90.55 (↓0.13)	62.78 (↑0.29)	93.23 (↓0.24)	0.76	0.93	94.85 (↓0.11)	93.26 (↓0.06)
4	+IM-CBAM	63.39 (↑0.26)	91.40 (↑0.85)	62.95 (↑0.17)	93.88 (↑0.65)	0.75	0.94	95.25 (↑0.40)	94.50 (↑1.24)
5	+Fourth head	64.26 (↑0.87)	93.47 (↑2.07)	63.50 (↑0.55)	94.97 (↑1.09)	0.76	0.95	95.85 (↑0.30)	94.65 (↑0.15)

**Table 4:** Comparison with results of advanced models.

No.	Model	AP (%)		Recall (%)		Params(M)	fps
		Bike	Car	Bike	Car		
1	Baseline	62.68	90.34	61.16	90.69	8.94	69.19
5	Ours	**64.26**	93.47	**63.50**	**94.97**	9.77	28.7
6	SSD	45.41	90.24	10.26	94.61	24.28	**124.17**
7	Faster-RCNN	0.19	57.61	0.95	78.37	28.33	24.37
8	YOLOv3	60.45	88.37	60.18	85.80	61.55	25.55
9	YOLOv4	64.20	73.55	61.12	71.35	63.96	18.42
10	YOLOv5	59.16	93.16	58.05	94.20	**7.08**	70.85
11	YOLOv7	62.39	**94.13**	61.05	94.53	37.22	25.74
12	YOLOv8	50.98	**94.68**	41.77	94.12	11.14	**128.46**

**Table 5:** Results of trajectory extraction.

Performances	Test Video #1		Test Video #2		Test Video #3	
	Car	Bike	Car	Bike	Car	Bike
Ground truth	14	116	14	102	25	37
True positive	13	95	13	87	23	27
False negative	1	21	1	15	2	10
False positive	1	13	1	10	1	5
Recall	92.86%	81.90%	92.86%	85.29%	92.00%	72.97%
Precision	92.86%	87.96%	92.86%	89.69%	95.83%	84.38%

## Data Availability

The data that support the findings of this study are available from the corresponding author upon reasonable request.
